# Aqueous Date Fruit Efficiency as Preventing Traumatic Brain Deterioration and Improving Pathological Parameters after Traumatic Brain Injury in Male Rats 

**DOI:** 10.22074/cellj.2016.4570

**Published:** 2016-08-24

**Authors:** Hamze Badeli, Nader Shahrokhi, Mahdieosadat KhoshNazar, Majid Asadi-Shekaari, Mohammad Shabani, Hassan Eftekhar Vaghefi, Mohammad Khaksari, Mohsen Basiri

**Affiliations:** 1 Department of Anatomical Sciences, Afzali Pour Medical Faculty, Kerman University of Medical Sciences, Kerman, Iran; 2 Physiology Research Center, Neuropharmacology Institute, Kerman University of Medical Sciences, Kerman, Iran; 3 Neuroscience Research Center, Neuropharmacology Institute, Kerman University of Medical Sciences, Kerman, Iran; 4 Department of Physiology, Afzali Pour Medical Faculty, Kerman University of Medical Sciences, Kerman, Iran

**Keywords:** Brain Injury, Brain Edema, Intracranial Pressure

## Abstract

**Objective:**

Following traumatic brain injury, disruption of blood-brain-barrier and consequent brain edema are critical events which might lead to increasing intracranial pressure (ICP), and nerve damage. The current study assessed the effects of aqueous date fruit extract (ADFE) on the aforementioned parameters.

**Materials and Methods:**

In this experimental study, diffused traumatic brain injury (TBI) was generated in adult male rats using Marmarou’s method. Experimental groups include two pre-treatment (oral ADFE, 4 and 8 mL/kg for 14 days), vehicle (distilled water, for 14 days) and sham groups. Brain edema and neuronal injury were measured 72 hours after TBI. Veterinary coma scale (VCS) and ICP were determined at -1, 4, 24, 48 and 72 hours after TBI. Differences among multiple groups were assessed using ANOVA. Turkey’s test was employed for the ANOVA post-hoc analysis. The criterion of statistical significance was sign at P<0.05.

**Results:**

Brain water content in ADFE-treated groups was decreased in comparison with the TBI+vehicle group. VCS at 24, 48 and 72 hours after TBI showed a significant increase in ADFE groups in comparison with the TBI+vehicle group. ICP at 24, 48 and 72 hours after TBI, was decreased in ADFE groups, compared to the TBI+vehicle. Brain edema, ICP and neuronal injury were also decreased in ADFE group, but VCS was increased following on TBI.

**Conclusion:**

ADFE pre-treatment demonstrated an efficient method for preventing traumatic brain deterioration and improving pathological parameters after TBI.

## Introduction

Traumatic brain injury (TBI) is one of the main causes of death and neurological disability among adolescent individuals worldwide (1). TBI produces an inflammatory reaction that is usually accompanied with intense apoptosis in different areas of the brain (2). The insult activates an incursion of macrophages into the crashed area, producing much of the inflammation and edema associated with brain damage. The cytotoxic events can directly affect patient outcome after TBI, which can be further exacerbated by uncontrolled intracranial pressure (ICP) (3), caused by an increase in brain water content (4). Uncontrolled ICP can produce greater secondary damage through ischemia (5) and increase mortality caused by hernia of the brain (6). Up to now, TBI treatment methods have been concentrated on the reduction of ICP and post-oxidative stress, but an effective pharmacological treatment remains to be found. Dates are widely used in order to helping people who are suffering from different disorders including memory disturbances, paralysis, inflammation and etc. (7). 

Aqueous date fruit extract (ADFE) might be interesting since they have been recently known as promising neuro-protective agents in some model of neuro-degeneration (7). Latest findings recommend that anti-oxidant agents might exert neuroprotective effects which may be promising in therapy. The importance of dates in human nutrition comes from its rich composition of carbohydrates, dietary fibers, salts and minerals, fatty acids, vitamins, amino acids, and proteins. In many ways, dates may be considered as a more or less ideal food (8). In addition, dates possess many valuable properties such as anti-oxidant (9), anti-bacterial (10), and anti-inflammatory (11). According to recent study by Asadi-Shekaari et al. (12), ADFE significantly inhibited neuronal injury induced by focal cerebral ischemia. Also they concluded that the efficacy of ADFE in focal cerebral ischemia is presumably due to its anti-oxidant property. 

Iranian date fruit is renowned for the presence of many classes of bioactive ingredients including polyphenols especially phenolic acids, lignans, flavonoids, tannins, carotenoids, and many others (13). Owing to its high nutritive values and potential health promoting activities, date fruit may be considered as potential candidate for the development of functional food (14). 

Here, using a rodent form of diffuse TBI, we addressed that ADFE administration before trauma caused a reduction in ICP, brain edema, neurologic outcome and neuronal injury in male rat. 

## Materials and Methods

**Fig.1:**
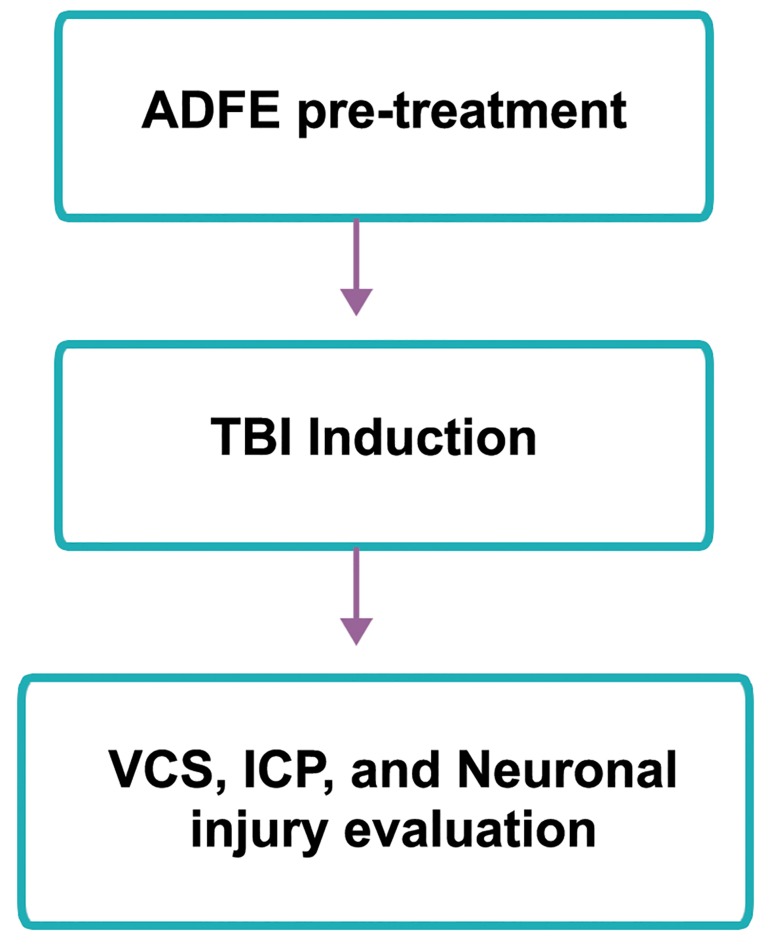
Flowchart diagram of research project. ADFE; Aqueous date fruit extract, TBI; Traumatic brain injury, VCS; Veterinary coma scale, and ICP; Intracranial pressure.

The approval of this experimental research study has been confirmed by Ethics Committee for the Animal Experimental Protocols of Kerman University of Medical Sciences (EC/KNRC/91-2). Adult male Albino N Mary rats (weighing 250320 g) were housed in an air-conditioned room in Afzali-Pour Medical Faculty, Kerman University of Medical Sciences (Kerman, Iran), at 2225˚C, with a 12 hours light: 12 hours dark cycle and free access to food and water. Animals were divided into four groups of sham, TBI+vehicle, low-dose ADFE (4 mL/kg), and high-dose ADFE (8 mL/kg). The number of animals in each group was 21. Each group was divided into three subgroups (n=7) for measuring brain water content and neurologic score (subgroup 1), ICP (subgroup 2), and neuronal injury (subgroup 3). Figure 1 represents schematic diagram of research procedure. 

## Experimental groups

The subsequent groups were established, including group 1: sham group, consisted of healthy rats undergoing procedure of brain trauma preparation but were not exposed to brain trauma; group 2: TBI+ADFE4 comprised of rats that were exposed to brain trauma and received orally 4 mL/kg ADFE for 14 days prior to TBI; group 3: TBI+ADFE8 comprised of rats that were exposed to brain trauma and received orally 8 mL/kg ADFE for 14 days prior to TBI; group 4: TBI+vehicle consisted of rats that were exposed to brain trauma and received orally distilled water for 14 days prior to TBI. 

## Marmarou’s rat acceleration-impact model 

The TBI diffusion was induced by the Marmarou’s method (15), using a TBI induction device made by Department of Physiology, Kerman University of Medical Sciences. The protocol was as follows: a 250 g weight was dropped from a 2 meters height on the head of the anesthetized (chloral hydrate, 400 mg/kg) rat animal while a metal disc (stainless steel, 10 mm in diameter, 3 mm thick) was attached to the animal’s skull. Control (sham-operated) animals were anesthetized and had the steel disc attached to the skull, but they did not receive the weight drop. The animals were connected to a respiratory pump, Technical and scientific equipment (TSE) animal respiratory compact, Bad Homburg, Germany) following induction of the trauma. After recovery they were kept in separate cages (16). 

## Determination of brain edema

Edema was measured directly by assessing water content in the brain. The weight of wet tissue was measured firstly and then incubated in 70˚C in an incubator (Memmert, Germany) for 72 hours to evaporate the tissue water and dry. The brain was then weighed again and water content was calculated using the below formula: 

$$\mathrm{Brain water content (\%)}=[\frac{\left(\mathrm{wet weight-dry weight}\right)}{\mathrm{wet weight}}]\times 100$$

## Evaluation of intracranial pressure 

ICP was determined by using ICP monitoring system made by Mobin Kahroba Kimia Co. (Iran). The anesthetized animal (with 68% N2O+30%O_2_+2% halothane, Piramal Critical Care, USA) was placed in streotax instrument in the way that the head was placed in the middle of sagital plane and the anterior-posterior point was located at about midpoint between the occipital crest and the lambda suture. After indenting cistern magna area, a needle (No. 20) connecting to E50 tube of ICP monitoring system was entered into the 5 mm depth of cisterna magna area and passed Dura to transfer the pressure to the transducer. By recording system (AD Instrument, Pty Ltd, Australia) the pressure was recorded before the trauma induction, and at 4 and 24 hours after TBI (17). 

## Evaluation of neurological outcomes

According to veterinary coma scale (VCS), the range of neurological score was determined between 3-15. This range was based on the sum of 3 parts: motor function (score range 1-8), eye function (score range 1-4), and respiration (score range 1-3). Based on VCS criteria, higher and lower scores represent better and worse neurological outcomes, respectively. In the present study, the outcomes were measured 1 hour before trauma induction and measurements were continued at 4, 24, 48 and 72 hours after TBI (18). 

## FluoroJade staining

At 72 hours of post-TBI, animals underwent ICP with saline (0.9% NaCl in distilled water) and 4% paraformaldehyde (Sigma, USA), subsequent to which the brain was extracted. The right hemisphere was processed for FJ histochemistry and the left one for Nissl staining. FJ histochemistry was carried out as previously described (19). In brief, 40 μm coronal sections containing the sensorimotor cortex were washed in 85% ethanol (Razi, Iran) with 1% NaOH, (Merck, Germany) 75% ethanol in distilled water. Then, tissue sections were washed in 0.06% KMnO_4_ (Sigma,USA)for 15 minutes and followed by rinsing again with distilled water. After tissue processing, they were incubated for 15 minutes in FJ solution (1 mL dilutedin 99 mL 0.1% acetic acid, Merck, Germany). Nuclei were afterwards stained by 4´-6-diamidino-2-phenylindole (DAPI, Merck, Germany) to determine total cells. 

## Nissl staining

For quantitative analysis, three days after TBI, the brains (left hemisphere) were processed according to standard histological methods. Paraffinized brains were cut into 5 μm sections on a rotary microtome and the sections were stained with Cresyl fast violet (Nissl method). Neuronal damage was then estimated for each animal as the rate of degenerated pyramidal neurons quantity to that of both surviving and degenerated in three distinct areas of the cortex in coronal sections (12). 

## Preparation of aqueous date fruit extract

To prepare ADFE, fresh ripe dates (Bam type) were prepared from Bam city, Kerman, Iran. The seed was removed and 100 g of date was immersed in 1000 mL/distilled water for 48 hours at 4˚C. It was then mixed thoroughly in a mechanical set and subsequently the mixture was centrifuged at 4000 rpm, 4˚C for 15 minutes. Following sedimentation, the supernatant part was used for gavages. 

## Chemical analysis of aqueous date fruit extract

The analytical gas chromatography (GC, Intertek, UK) was carried out on an Agilent GC-mass spectrometry (GC-MS) system (Intertek, UK), equipped with a Chrompack (5%-Phenyl)-methylpolysiloxane (5 ms) 30 mm×0.25 mm×0.25 μm film thickness capillary column. The chromatography was recorded with integrator. The column temperature was programmed from 60 (for 5 minutes), to 280˚C at a rate of 3˚C per minute and held at 280˚C for 15 minutes. The injector and detector temperatures were programmed at 220 and 280˚C, respectively. Helium was used as carrier gas, while flow rate and split ratio were respectively 1 mL/minutes and 1:10. MS details include ionization energy=70 eV, emission=200 μA, mass range=35-650 Da, scan time=1.25 seconds, scan rate (amu/seconds) =500.0 and scans/seconds=0.7974. 

All compounds were identified by comparison of their retention times (RT) and mass spectra with those of authentic samples and/or using national institute of standards and technology (NIST)/national bureau of standards publications (NBS), NIST02, Wiley 575, Wiley 6, libraries spectra and through international literatures (20). 

## Statistical analysis

Statistical analysis was performed with SPSS adopted for Windows. All data were represented as mean ± SEM. Differences among multiple groups were assessed using ANOVA. Turkey’s test was employed for the NOVA post-hoc analysis. Statistical significance criterion was signed at a P<0.05. 

## Results

### Brain edema

**Fig.2:**
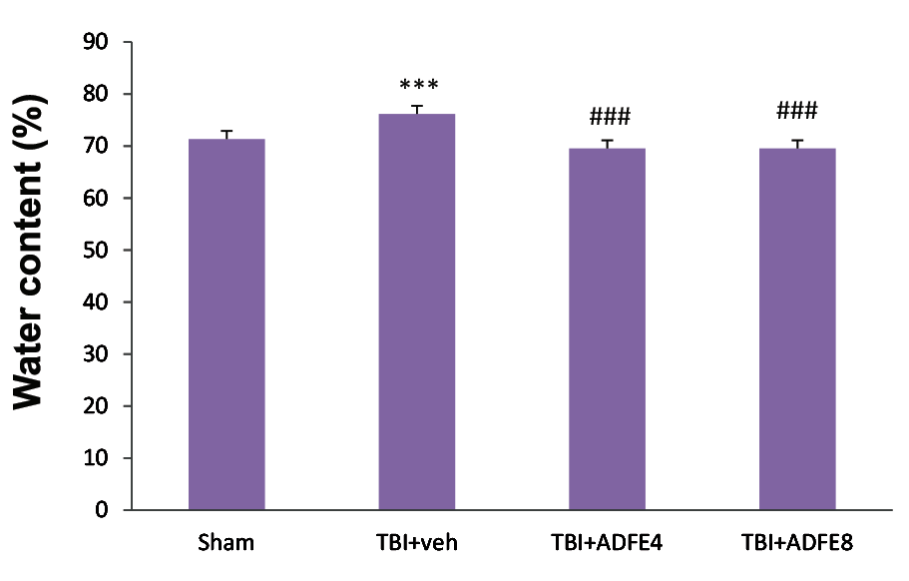
The effect of two ADFE dosages on brain water content 72 hours after inducing TBI in male rats. Data are presented as mean ± SEM, n= 7/groups. ***; Significant difference between sham groups (P<0.001), ###; Significant difference compared to TBI+vehicle group (P<0.001), TBI; Traumatic brain injury, ADFE; Aqueous date fruit extract, and TBI+veh; TBI+vehicle.

The water content of the brain in the different groups, at 72 hours of post TBI has been shown in the Figure 2. This figure shows that the brain water content in TBI+vehicle (76.15%) group is significantly more than sham (71.34%, P<0.001).On the other hand, the brain water content in TBI+ADFE4 (69.54%) and TBI+ADFE8 (69.50%) groups were significantly less than that of TBI+vehicle group (P<0.001); whereas there was no significant difference among TBI+ADFE4 or ADFE8 groups (Fig.2). 

### Intracranial pressure measurements

**Fig.3:**
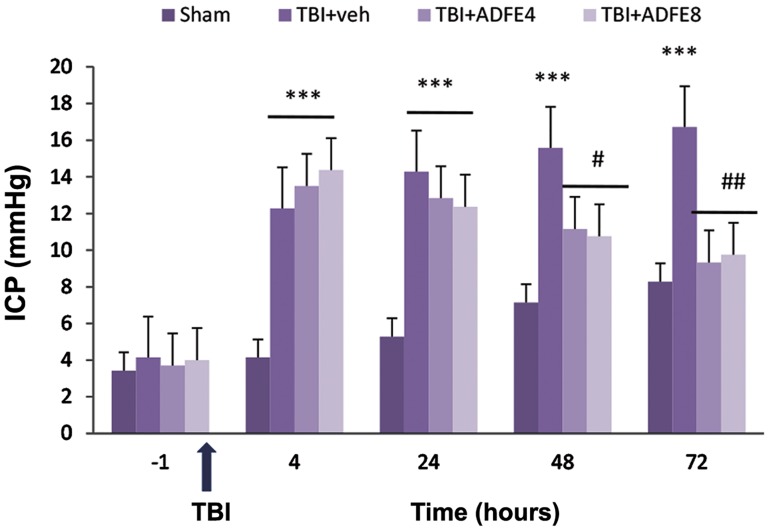
The effect of two ADFE dosages on ICP (mmHg) after inducing TBI in male rats. Data are presented as mean ± SEM, n = 7/group. ***; Significant difference between TBI groups and sham group (P<0.001). #, ##; Respectively represent P<0.05 and P<0.01 significant difference between TBI+ADFE4 and TBI+ADFE8 groups with TBI+vehicle, TBI; Traumatic brain injury, ADFE; Aqueous date fruit extract, ICP; Intracranial pressure, and TBI+veh; TBI+vehicle.

Figure 3 illustrates the ICP alterations in all traumatic groups at different times after TBI. As seen in this Figure, 1 hour before TBI, there was no significant difference among these groups, with regard to ICP. The induction of trauma caused increase in ICP. Thus, 4 and 24 hours after trauma, the ICP was increased in TBI+vehicle, as well as both TBI+ADFE4 and TBI+ADFE8 groups compared to sham group (P<0.001); although there was no significant difference in ICP between the TBI+vehicle and TBI+ADFE groups. TBI+ADFE groups showed a significant reduction in ICP at 24, 48 and 72 hours after TBI (P<0.01, Fig.3). 

### Neurological scores evaluations

**Fig.4:**
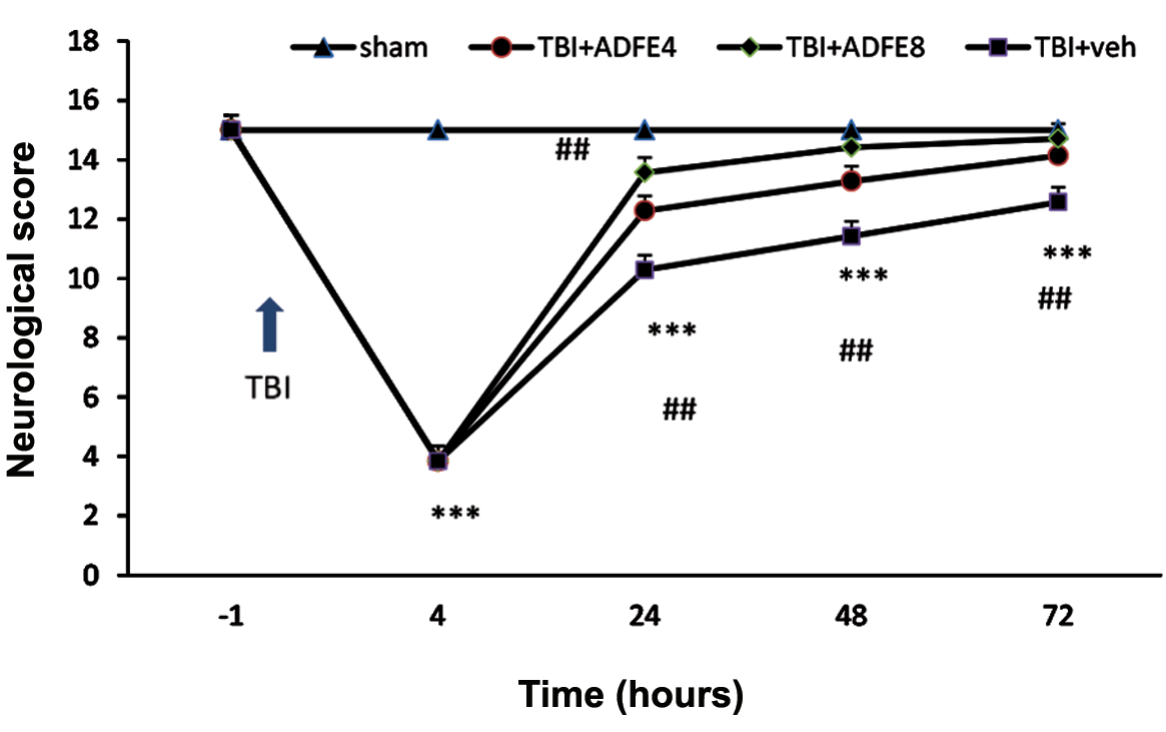
The effect of two ADFE dosages on neurological scores after inducing TBI in male rats. ***; Significant difference compared to sham group (P<0.001). Neurological scores were increased in the pre-treated TBI groups with ADFE, in comparison with TBI+vehicle group, ##; P<0.01. Data are presented as mean ± SEM, n=7/group, TBI; Traumatic brain injury, ADFE; Aqueous date fruit extract, and VCS; Veterinary coma scale.

Neurological scores (VCS) alterations have been shown in the experimental groups of animals in the Figure 4. This figure shows that at1 hour before TBI induction there was no significant difference between the groups, although at 4 hours after TBI, a marked decrease in VCS score in the TBI+vehicle, TBI, and both ADFE-treated groups were observed in comparison with the sham (P<0.001). Our data, after 24, 48, and 72 hours of time-frame have shown a significant increase in VCS in both treated groups (TBI+ADFE4 and TBI+ADFE8) compared to TBI+vehicle (P<0.05 and P<0.01 respectively, Fig.4). 

### Neuronal degeneration 

**Fig.5:**
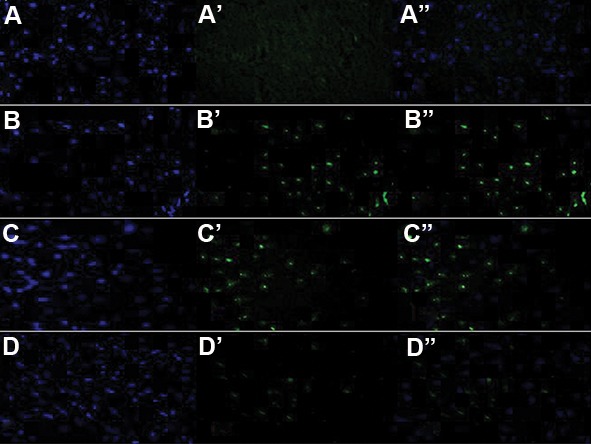
Sections containing the cerebral cortex were double-labeled with A., B., C., D. DAPI and A'., B'., C'., D'. FJ or A''., B''., C''., D''. Merged together to show degeneration of neurons. FluoroJade staining of these sections demonstrate the extensive neuronal damage that has occurred in B. TBI+vehicle group, compared to A. Sham group. Pre-treatment with ADFE decreased neurodegeneration in cer- ebral cortex as observed in C and D. DAPI; 4'-6-diamidino-2-phenylindole, TBI; Traumatic brain injury, and ADFE; Aqueous date fruit extract.

Neuronal degeneration after TBI was assessed by FJ staining at 72 hours post-trauma. Compared to sham (Fig.5A), intense FJ-labeled neuronal perikarya were detected in the cortex (Fig.5B). While,in all regions, FJ labeled cell quantity was significantly reduced as a result of pre-treatment with ADFE (Fig.5C,D). 

Compared to the treated TBI animals with vehicle, analyses of the injured cortex in pre-treated animals with ADFE demonstrated a significant reduction in neuronal injury. 

### Neuronal counting

**Fig.6:**
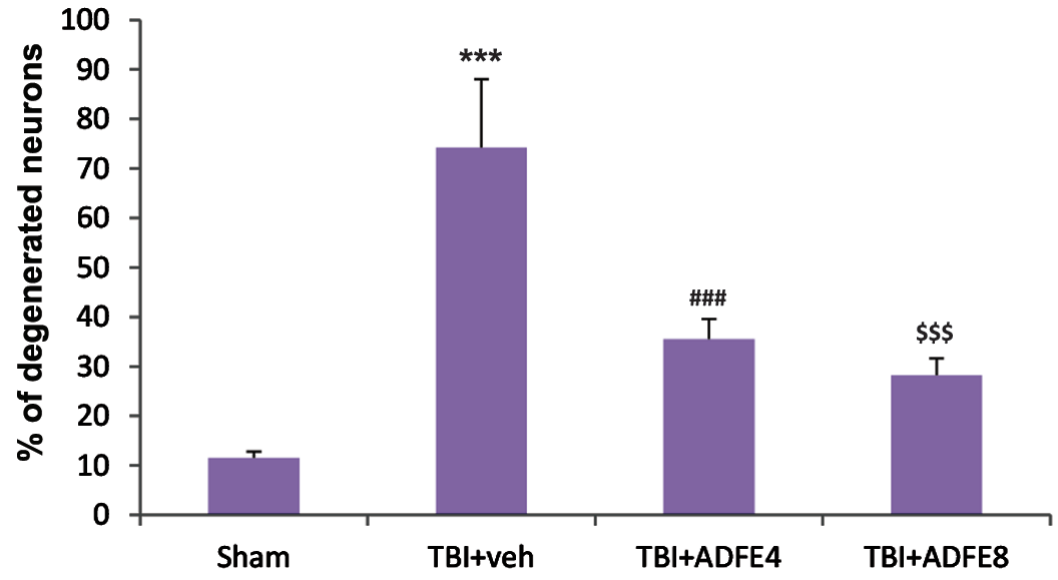
Neuronal injury in cerebral cortex 72 hours after TBI was assessed using Nissl’s staining. Results were expressed as mean ± SEM. ***; Significant difference compared to sham group (P<0.001), ###; Significant difference compared to TBI+vehicle group (P<0.001), $$$; Significant difference compared to TBI+vehicle group (P<0.001), TBI; Traumatic brain injury, ADFE; Aqueous date fruit, and TBI+veh; TBI+vehicle.

According to the obtained data, TBI induced severe neurodegeneration in the cerebral cortex (74.25%). Pre-treatment with different doses (4 and 8 ml/kg) of ADFE significantly decreased neurnodegeneration in the cerebral cortex (35.5 and 28.25% respectively, Fig.6). 

### Aqueous date fruit extract chemical analysis 

ADFE analytic assay showed that major compounds of the used extract are phenolic compounds such as phenolic acids (protocatechuic, vanilic, pcomaric, o-coumaric, ferulic and synergic), flavonoids, anthocyanins2-Oxoglutaric acid, phloroglucinol, and pterin-6-carboxylic acid. 

## Discussion

In the present study, in order to ascertain the neuroprotective effects of ADFE diet, we investigated the role of ADFE diet in the development of brain edema, ICP, and neuronal degeneration after TBI. The main finding was acquired in this study implicated that ADFEs (4, 8 ml/kg/day, oral administration) reduced brain edema, while ICP improved neurological scores and prevented increased neuronal degeneration at post-TBI in male rats. 

The results of experimental studies have revealed that blood-brain barrier (BBB) breakdown and elevating endothelium permeability, after brain trauma, can cause brain edema and eventually ICP increase (21). Brain edema is one of the main factors contributing to cell injury at post-TBI (22). It is generally believed that edema causes a rise in ICP, which contributes significantly to neuronal cell injury (23), and also increases the mortality and morbidity rates in TBI patients (24). It seems that oxidative stress has a key role in postTBI neuronal injury (25). It has been recognized that accumulation of oxygen-derived free radicals in vessels has a key role in the molecular cascade involved in BBB breakdown (26). Moreover, secondary BBB disruption is also due to the development of inflammatory mechanisms (21). 

The obtained data from the current study showed that pre-treatment with ADFE significantly ameliorates brain edema 72 hours after TBI. This effect may be due to anti-inflammatory and antioxidant effects of ADFE. 

The present study has also showed that different dosages of ADFE can be efficient in decreasing post-TBI intracranial pressure in a way that immediately after TBI, ICP in vehicle-treated and ADFE groups was significantly increased, compared to the sham group. ICP increase, occurred one hour after trauma, was continued for 72 hours in all groups (16). Although a number of studies reported no ICP increase, after TBI (27,28). Sham-operated animals, like traumatic animals, showed a relative increase of ICP at different posttraumatic hours. The mechanism of ICP increase in sham-operated group is not clear yet; however, inserting the probe of ICP assessment device could further clarify the cause of ICP increase in different groups (29). It has been recognized that ICP increase induces cerebral perfusion pressure (CPP) and prohibits cerebral blood flow (CBF), which might subsequently result in secondary cerebral ischemia (30). In fact, possible causes of ICP increase in traumatic groups could be secondary injuries due to the brain compartments and contusion damages (31), brain blood volume increase or constriction of meningeal layers surrounding the brain (32) as well as CBF decline (33). 

Alternatively, TBI produces a considerable inflammatory reaction that is generally accompanied by intense apoptosis in different areas of the brain (2). The results of our study showed that TBI caused severe neuronal degeneration in the brain cortex, whilepre-treatment with ADFE ameliorated neurodegeneration in brain cortex. The cerebroprotective effect of ADFE has previously confirmed (34). They reported that the positive effects of the extract are dependent upon its antiinflammatory and anti-oxidant properties. 

Neurologic scores of ADFE pre-treated groups at different post-TBIs were higher compared to the vehicle group. It has been reported that low neurologic scores are associated with vasoconstriction and cerebral hypoperfusion (35). Cerebral hypoperfusion after TBI is related to reduction in tissue oxygen (36). A diet rich potassium enhances respective serum potassium, causing endotheliumdependent vasodilation by hyperpolarizing the endothelial cell through stimulation of sodium pump and opening potassium channels (37). ADFE is an excellent source of potassium with very low sodium content. On the other hand, one of the beneficial components of ADFE is melatonin (38) that may be involved in this protective effect of ADFE. Recently, it was shown that melatonin can increase neurologic scores after TBI in male rats (17). Although the precise mechanism of this protective effect is not yet known, high potassium and melatonin content levels could be an explanation for this effect. 

ADFE pre-treatment for 14 days prior to TBI is effective in decreasing brain edema, ICP, and neuronal degeneration, as well as in improving neurological scores. These positive properties might be due to its potential anti-oxidant and antiinflammatory effects. As mentioned above, ADFE has strong anti-oxidant activity. The anti-oxidant effect of ADFE is mostly attributed to the phenolic compounds (hydrophilic antioxidants) such as phenolic acids (protocatechuic, vanilic, p-comaric, o-coumaric, ferulic and synergic), flavonoids, and anthocyanins presented in it (14). Some of the novelrecognized anti-inflammatory and antioxidant componentsin our utilized extract consist of 2-Oxoglutaric acid, 2-Methyl-1,3-Cyclopentanedione, phloroglucinol, pterin-6-carboxylic acid. It is worthy to mention that the phenolic compounds are identified to act as anti-oxidants or by the mechanisms other than their anti-oxidant action (39). These components can inhibit production of reactive oxygen species (ROS) by inhibiting several ROS producing enzymes, and by chelating trace metals and inhibiting phospholipase A2 and C (40). ADFE may also increase the endogenous anti-oxidant enzyme activity by providing metal ions in the form of dietary minerals such as Zn, Se, and Mg as cofactors in several anti-oxidant enzymes (9). 

## Conclusion

Based on our data, the ADFE diethad neuroprotective function. Indeed, ADFE diet inhibited the TBI-induced increase in brain edema, ICP, neuronal degeneration, and improved neurological outcomes. This may suggest that dates consumption has beneficial effects on reducing the damage caused by trauma; nevertheless, there is a need for the further studies for revealing the signalling pathways involved in the neuroprotective function of ADFE diet. 
